# 2′-Acetonaphthone

**DOI:** 10.1107/S1600536812039554

**Published:** 2012-10-13

**Authors:** Ibukun O. Shotonwa, René T. Boeré

**Affiliations:** a Department of Chemistry and Biochemistry, University of Lethbridge, Lethbridge, AB, Canada T1K3M4

## Abstract

In the structure of the title compound [systematic name: 1-(naphthalen-2-yl)ethanone], C_12_H_10_O, the acetyl group is approximately coplanar with the naphthalene ring with a C_ar_—C_ar_—C=O torsion angle of 5.8 (2)°. In the crystal, the mol­ecules are packed in a classic herringbone arrangement typical for aromatic polycycles such as penta­cene. They are also linked by weak end-to-end C—H⋯O interactions along the *ac* diagonal.

## Related literature

For synthesis details, see: Bassilios & Salem (1952 ▶). For related structures, see: Kemperman *et al.* (2000 ▶); Mattheus *et al.* (2001 ▶); Miyake *et al.* (1998 ▶). For a description of the Cambridge Structural Database, see: Allen (2002 ▶).
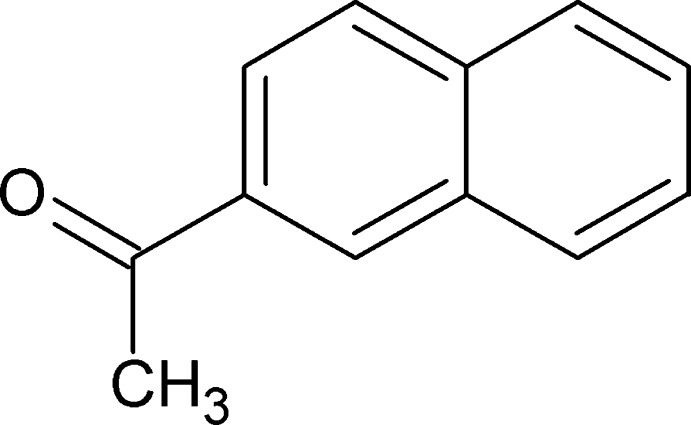


## Experimental

### 

#### Crystal data

C_12_H_10_O
*M* *_r_* = 170.20
Monoclinic, 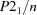

*a* = 5.9875 (5) Å
*b* = 7.4025 (7) Å
*c* = 20.2778 (18) Å
β = 93.747 (1)°
*V* = 896.84 (14) Å^3^
*Z* = 4
Mo *K*α radiation
μ = 0.08 mm^−1^
*T* = 173 K
0.3 × 0.25 × 0.2 mm

#### Data collection

Bruker APEXII CCD area-detector diffractometer
Absorption correction: multi-scan (*SADABS*; Bruker, 2008 ▶) *T*_min_ = 0.701, *T*_max_ = 0.746
12546 measured reflections
2089 independent reflections
1840 reflections with *I* > 2σ(*I*)
*R*_int_ = 0.019

#### Refinement

*R*[*F* ^2^ > 2σ(*F* ^2^)] = 0.040
*wR*(*F* ^2^) = 0.118
*S* = 1.08
2089 reflections
119 parameters
H-atom parameters constrained
Δρ_max_ = 0.25 e Å^−3^
Δρ_min_ = −0.23 e Å^−3^

### 

Data collection: *APEX2* (Bruker, 2008 ▶); cell refinement: *SAINT-Plus* (Bruker, 2008 ▶); data reduction: *SAINT-Plus*; program(s) used to solve structure: *SHELXS97* (Sheldrick, 2008 ▶); program(s) used to refine structure: *SHELXTL* (Sheldrick, 2008 ▶); molecular graphics: *Mercury* (Macrae *et al.*, 2008 ▶); software used to prepare material for publication: *publCIF* (Westrip, 2010 ▶).

## Supplementary Material

Crystal structure: contains datablock(s) I, global. DOI: 10.1107/S1600536812039554/hg5250sup1.cif

Structure factors: contains datablock(s) I. DOI: 10.1107/S1600536812039554/hg5250Isup2.hkl

Supplementary material file. DOI: 10.1107/S1600536812039554/hg5250Isup3.cml

Additional supplementary materials:  crystallographic information; 3D view; checkCIF report

## Figures and Tables

**Table 1:** Hydrogen-bond geometry (Å, °)

*D*—H⋯*A*	*D*—H	H⋯*A*	*D*⋯*A*	*D*—H⋯*A*
C8—H8⋯O1^i^	0.95	2.65	3.324 (1)	129

Symmetry code: (i) 
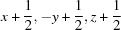
.

**Fig. 1.**
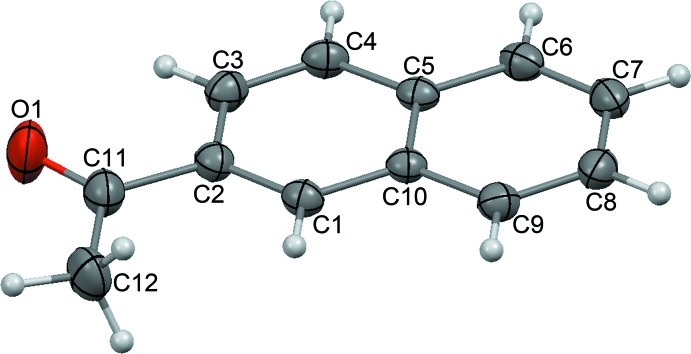
Molecular structure of (I) drawn with displacement elipsoids at the 50% probability level and showing the atom numbering scheme.

**Fig. 2.**
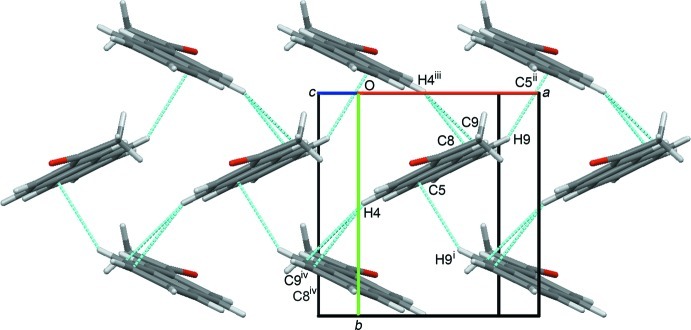
An extended packing diagram viewed down the c* direction, showing the "herringbone" edge-to-face packing arrangements. Only atoms involved in short contacts to neighbouring atoms are labelled [Sym. codes: (i) -x + 3/2, y + 1/2, -z + 1/2; (ii) -x + 3/2, y - 1/2, -z + 1/2; (iii) -x + 1/2, y - 1/2, -z + 1/2; (iv) -x + 1/2, y + 1/2, -z + 1/2]. The O1···H8—C8 H-bonds are not shown but are oriented along the ac diagonal (approximately perpendicular to the page).

## supplementary crystallographic information

### Comment

2'-Acetonaphthone, (I), is an important example of an aromatic ketone that can be prepared by a classical Friedel-Crafts acylation reaction (Bassilios & Salem, 1952) and is commercially available from many suppliers. A search of the Cambridge Structural Database (Allen, 2002; WebCSD August 2012) returned only two previous crystal structures for (I), in both of which this molecule functions as a guest within an organic host framework. In the structure reported by Kemperman *et al.* (2000; refcode MEGXUR), (I) is found as a disordered inclusion compound along with four water molecules in a clathrate formed by two cephradine molecules. The cages formed by this cephalosporin antibiotic were shown to be quite flexible and fit guests of differing size, in part by also incorporating varying numbers of hydrogen-bonded water molecules. This adaptability of the host lattice has been described as permitting "induced fitting" of guest molecule(*s*). The cephradine host molecules fully surround their guests and keep individual molecules of (I) separated by the *b* axis distance of 7.1965 (3) Å. In the second example (refcode: NECPUG), (I) forms into π-stacks which fill channels that run along the *c* axis of a lattice formed from the modified bile acid derivative 3-epiursodeoxycholic acid (Miyake *et al.*, 1998). The average separation of molecules of (I) along these channels is 3.51 Å, just 0.1 Å greater than the sums of the van der Waals radii of two carbon atoms. Both of these structures for (I) have very poor precision in the interatomic distances with mean s.u. of 0.01 Å.

We have therefore determined the crystal structure at 173 K of pure (I). Fig. 1 displays the molecular structure as found in the crystal lattice. The acetyl group is approximately co-planar with the naphthalene ring and the carbonyl oxygen is *anti* to the ring with the torsion angle C1-C2-C11-O1 174.8 (1)°. By comparison, in NECPUG the oxygen atom is in the *syn* position. The disorder in MEGXUR precludes a definitive conformational assignment, but the major component appears to have the oxygen *anti* as in (I). It is instructive to compare the bond distances determined for pure (I) with those determined in the host lattices. The high-accuracy structure reported here may also be used to define rigid templates as an aid in refining future inclusion compounds of (I).

In contrast to the host–guest complexes MEGXUR, which has isolated molecules of (I), and NECPUG with π-stacked (I), the crystal packing of pure (I) is of the herringbone 2-D edge-to-face type (Figure 2). This arrangement of crystal packing is reminiscent to that found in pentacene as determined at 90 K (Mattheus *et al.*, 2001). Unlike pentacene, molecules of (I) are also linked by weak end-to-end by C8-H8···O1 intermolecular interactions (Table 1).

### Experimental

A sample of (I) was prepared by the method of Bassilios and Salem (1952).

### Refinement

Hydrogen atoms attached to carbon were treated as riding, with C—H = 0.98 Å and *U*_iso_(H) = 1.5*U*_eq_(C) for methyl and C—H = 0.95 Å and *U*_iso_(H) = 1.2*U*_eq_(C) for aromatic H atoms.

### Crystal data

**Table:**

C_12_H_10_O	*F*(000) = 360
*M**_r_* = 170.20	*D*_x_ = 1.261 Mg m^−^^3^
Monoclinic, *P*2_1_/*n*	Melting point: 326.7 K
Hall symbol: -P 2yn	Mo *K*α radiation, λ = 0.71073 Å
*a* = 5.9875 (5) Å	Cell parameters from 7818 reflections
*b* = 7.4025 (7) Å	θ = 2.8–27.6°
*c* = 20.2778 (18) Å	µ = 0.08 mm^−^^1^
β = 93.747 (1)°	*T* = 173 K
*V* = 896.84 (14) Å^3^	Block, colourless
*Z* = 4	0.3 × 0.25 × 0.2 mm

### Data collection

**Table:**

Bruker APEXII CCD area-detector diffractometer	2089 independent reflections
Radiation source: fine-focus sealed tube, Bruker D8	1840 reflections with *I* > 2σ(*I*)
Graphite monochromator	*R*_int_ = 0.019
Detector resolution: 66.06 pixels mm^-1^	θ_max_ = 27.6°, θ_min_ = 2.0°
φ and ω scans	*h* = −7→7
Absorption correction: multi-scan (*SADABS*; Bruker, 2008)	*k* = −9→9
*T*_min_ = 0.701, *T*_max_ = 0.746	*l* = −26→26
12546 measured reflections	

### Refinement

**Table:**

Refinement on *F*^2^	Primary atom site location: structure-invariant direct methods
Least-squares matrix: full	Secondary atom site location: difference Fourier map
*R*[*F*^2^ > 2σ(*F*^2^)] = 0.040	Hydrogen site location: inferred from neighbouring sites
*wR*(*F*^2^) = 0.118	H-atom parameters constrained
*S* = 1.08	*w* = 1/[σ^2^(*F*_o_^2^) + (0.0634*P*)^2^ + 0.1624*P*] where *P* = (*F*_o_^2^ + 2*F*_c_^2^)/3
2089 reflections	(Δ/σ)_max_ < 0.001
119 parameters	Δρ_max_ = 0.25 e Å^−^^3^
0 restraints	Δρ_min_ = −0.23 e Å^−^^3^

### Special details

**Table:**

Experimental. A crystal coated in Paratone (TM) oil was mounted on the end of a thin glass capillary and cooled in the gas stream of the diffractometer Kryoflex device.
Geometry. All e.s.d.'s (except the e.s.d. in the dihedral angle between two l.s. planes) are estimated using the full covariance matrix. The cell e.s.d.'s are taken into account individually in the estimation of e.s.d.'s in distances, angles and torsion angles; correlations between e.s.d.'s in cell parameters are only used when they are defined by crystal symmetry. An approximate (isotropic) treatment of cell e.s.d.'s is used for estimating e.s.d.'s involving l.s. planes.
Refinement. Refinement of *F*^2^ against ALL reflections. The weighted *R*-factor *wR* and goodness of fit *S* are based on *F*^2^, conventional *R*-factors *R* are based on *F*, with *F* set to zero for negative *F*^2^. The threshold expression of *F*^2^ > σ(*F*^2^) is used only for calculating *R*-factors(gt) *etc*. and is not relevant to the choice of reflections for refinement. *R*-factors based on *F*^2^ are statistically about twice as large as those based on *F*, and *R*- factors based on ALL data will be even larger.

### Fractional atomic coordinates and isotropic or equivalent isotropic displacement parameters (Å^2^)

**Table:**

	*x*	*y*	*z*	*U*_iso_*/*U*_eq_	
O1	0.29572 (15)	0.31555 (14)	−0.00905 (4)	0.0539 (3)	
C1	0.60105 (15)	0.27553 (12)	0.14913 (4)	0.0256 (2)	
H1	0.7348	0.2220	0.1356	0.031*	
C2	0.43438 (16)	0.32203 (13)	0.10228 (4)	0.0274 (2)	
C3	0.23586 (16)	0.40517 (13)	0.12238 (5)	0.0301 (2)	
H3	0.1214	0.4389	0.0901	0.036*	
C4	0.20800 (15)	0.43702 (13)	0.18753 (5)	0.0284 (2)	
H4	0.0745	0.4931	0.2001	0.034*	
C5	0.37603 (15)	0.38738 (12)	0.23698 (4)	0.0246 (2)	
C6	0.35241 (17)	0.41832 (13)	0.30521 (5)	0.0298 (2)	
H6	0.2199	0.4731	0.3191	0.036*	
C7	0.51965 (17)	0.36962 (14)	0.35120 (5)	0.0330 (2)	
H7	0.5016	0.3906	0.3968	0.040*	
C8	0.71788 (17)	0.28884 (14)	0.33168 (5)	0.0325 (2)	
H8	0.8322	0.2554	0.3641	0.039*	
C9	0.74625 (15)	0.25840 (13)	0.26613 (5)	0.0280 (2)	
H9	0.8809	0.2048	0.2533	0.034*	
C10	0.57636 (15)	0.30626 (12)	0.21724 (4)	0.0237 (2)	
C11	0.45374 (18)	0.28604 (15)	0.03043 (5)	0.0351 (3)	
C12	0.6697 (2)	0.21278 (19)	0.00735 (5)	0.0450 (3)	
H12A	0.6551	0.1955	−0.0407	0.067*	
H12B	0.7035	0.0967	0.0290	0.067*	
H12C	0.7910	0.2984	0.0187	0.067*	

### Atomic displacement parameters (Å^2^)

**Table:**

	*U*^11^	*U*^22^	*U*^33^	*U*^12^	*U*^13^	*U*^23^
O1	0.0509 (5)	0.0794 (7)	0.0299 (4)	0.0071 (5)	−0.0076 (4)	−0.0073 (4)
C1	0.0253 (4)	0.0241 (4)	0.0279 (5)	0.0005 (3)	0.0048 (3)	−0.0005 (3)
C2	0.0296 (5)	0.0268 (5)	0.0257 (5)	−0.0027 (4)	0.0024 (4)	0.0000 (3)
C3	0.0264 (5)	0.0310 (5)	0.0323 (5)	0.0004 (4)	−0.0022 (4)	0.0037 (4)
C4	0.0238 (4)	0.0264 (5)	0.0355 (5)	0.0024 (3)	0.0041 (4)	0.0015 (4)
C5	0.0246 (4)	0.0206 (4)	0.0291 (5)	−0.0024 (3)	0.0050 (3)	−0.0001 (3)
C6	0.0311 (5)	0.0280 (5)	0.0313 (5)	−0.0035 (4)	0.0090 (4)	−0.0030 (4)
C7	0.0386 (5)	0.0357 (5)	0.0254 (4)	−0.0101 (4)	0.0062 (4)	−0.0031 (4)
C8	0.0319 (5)	0.0355 (5)	0.0293 (5)	−0.0068 (4)	−0.0035 (4)	0.0047 (4)
C9	0.0250 (4)	0.0277 (5)	0.0312 (5)	−0.0004 (3)	0.0013 (4)	0.0032 (4)
C10	0.0237 (4)	0.0207 (4)	0.0268 (4)	−0.0017 (3)	0.0027 (3)	0.0014 (3)
C11	0.0404 (6)	0.0376 (6)	0.0270 (5)	−0.0032 (4)	0.0007 (4)	−0.0017 (4)
C12	0.0473 (6)	0.0594 (8)	0.0290 (5)	0.0020 (5)	0.0086 (4)	−0.0075 (5)

### Geometric parameters (Å, º)

**Table:**

O1—C11	1.2185 (13)	C6—C7	1.3712 (14)
C1—C2	1.3759 (13)	C6—H6	0.9500
C1—C10	1.4169 (12)	C7—C8	1.4086 (15)
C1—H1	0.9500	C7—H7	0.9500
C2—C3	1.4216 (13)	C8—C9	1.3697 (14)
C2—C11	1.4931 (13)	C8—H8	0.9500
C3—C4	1.3629 (14)	C9—C10	1.4181 (12)
C3—H3	0.9500	C9—H9	0.9500
C4—C5	1.4215 (13)	C11—C12	1.5046 (16)
C4—H4	0.9500	C12—H12A	0.9800
C5—C6	1.4186 (13)	C12—H12B	0.9800
C5—C10	1.4220 (12)	C12—H12C	0.9800
			
C2—C1—C10	121.04 (8)	C8—C7—H7	119.6
C2—C1—H1	119.5	C9—C8—C7	120.18 (9)
C10—C1—H1	119.5	C9—C8—H8	119.9
C1—C2—C3	119.51 (8)	C7—C8—H8	119.9
C1—C2—C11	122.01 (9)	C8—C9—C10	120.56 (9)
C3—C2—C11	118.47 (9)	C8—C9—H9	119.7
C4—C3—C2	120.69 (8)	C10—C9—H9	119.7
C4—C3—H3	119.7	C1—C10—C9	121.70 (8)
C2—C3—H3	119.7	C1—C10—C5	119.06 (8)
C3—C4—C5	120.88 (8)	C9—C10—C5	119.24 (8)
C3—C4—H4	119.6	O1—C11—C2	120.18 (10)
C5—C4—H4	119.6	O1—C11—C12	120.42 (10)
C6—C5—C4	122.35 (8)	C2—C11—C12	119.40 (9)
C6—C5—C10	118.84 (8)	C11—C12—H12A	109.5
C4—C5—C10	118.80 (8)	C11—C12—H12B	109.5
C7—C6—C5	120.39 (9)	H12A—C12—H12B	109.5
C7—C6—H6	119.8	C11—C12—H12C	109.5
C5—C6—H6	119.8	H12A—C12—H12C	109.5
C6—C7—C8	120.78 (9)	H12B—C12—H12C	109.5
C6—C7—H7	119.6		
			
C10—C1—C2—C3	1.13 (14)	C2—C1—C10—C9	179.74 (8)
C10—C1—C2—C11	−178.17 (8)	C2—C1—C10—C5	−0.33 (14)
C1—C2—C3—C4	−0.85 (14)	C8—C9—C10—C1	−179.72 (8)
C11—C2—C3—C4	178.47 (9)	C8—C9—C10—C5	0.34 (14)
C2—C3—C4—C5	−0.25 (15)	C6—C5—C10—C1	−179.82 (8)
C3—C4—C5—C6	−179.92 (9)	C4—C5—C10—C1	−0.75 (13)
C3—C4—C5—C10	1.04 (14)	C6—C5—C10—C9	0.11 (13)
C4—C5—C6—C7	−179.44 (8)	C4—C5—C10—C9	179.19 (8)
C10—C5—C6—C7	−0.40 (14)	C1—C2—C11—O1	174.03 (10)
C5—C6—C7—C8	0.25 (15)	C3—C2—C11—O1	−5.28 (16)
C6—C7—C8—C9	0.22 (15)	C1—C2—C11—C12	−5.85 (16)
C7—C8—C9—C10	−0.51 (15)	C3—C2—C11—C12	174.85 (10)

### Hydrogen-bond geometry (Å, º)

**Table:**

*D*—H···*A*	*D*—H	H···*A*	*D*···*A*	*D*—H···*A*
C8—H8···O1^i^	0.95	2.65	3.324 (1)	129

Symmetry code: (i) *x*+1/2, −*y*+1/2, *z*+1/2.
